# Newer treatment paradigm improves outcomes in the most common neurosurgical disease of the elderly: a literature review of middle meningeal artery embolization for chronic subdural hematoma

**DOI:** 10.1007/s11357-024-01173-5

**Published:** 2024-05-01

**Authors:** Luca H. Debs, Samantha E. Walker, Scott Y. Rahimi

**Affiliations:** Neurosurgery Department, Medical College of Georgia, Augusta, GA USA

**Keywords:** Chronic subdural hematoma, Middle meningeal artery, Embolization, Elderly, SDH, MMA

## Abstract

Chronic subdural hematoma (cSDH) is one of the most prevalent neurosurgical diseases, especially in the elderly. Yet, its incidence is predicted to increase further, paralleling the growth of the geriatric population. While surgical evacuation is technically straightforward, it is associated with significant morbidity and mortality. In fact, 30% of patients are expected to have hematoma recurrence and to need repeat surgical evacuation, and 20% of patients are expected to lose independence and require long-term care. A pathophysiology more complex than originally presumed explains the disappointing results observed for decades. At its core, the formation of microcapillaries and anastomotic channels with the middle meningeal artery (MMA) perpetuates a constant cycle resulting in persistence of hematoma. The rationale behind MMA embolization is simple: to stop cSDH at its source. Over the last few years, this “newer” option has been heavily studied. It has shown tremendous potential in decreasing hematoma recurrence and improving neurological outcomes. Whether combined with surgical evacuation or performed as the only treatment, the scientific evidence to its benefits is unequivocal. Here, we aimed to review cSDH in the elderly and discuss its more recent treatment options with an emphasis on MMA embolization.

## Introduction

Chronic subdural hematoma (cSDH) is a neurosurgical pathology with notable prevalence and morbidity among the geriatric population that is predicted to become the most common indication for cranial neurosurgical intervention [4, 11, 15, 56]. The estimated annual incidence in the general population is 13.5 cases per 100,000, but this estimate increases about tenfold in individuals 80 years and older [4, 39]. Risk factors contributing to the formation of cSDH involve cerebral atrophy coupled with altered parenchymal compliance, coagulopathy, dysregulation of the immune system, and head trauma [2, 14, 18, 36, 39, 41].

Presentation typically includes an insidious onset of non-specific symptoms such as headaches, confusion, and speech abnormalities. However, patients can more rarely develop motor deficits, sensory changes, and seizures, all without recall of an inciting injury [11, 14, 29, 68]. Computed tomography (CT) is the primary imaging modality for diagnosis. Observation with repeat clinical examination and serial imaging may be an option for asymptomatic patients, but surgical evacuation is the essential and effective intervention for symptomatic patients or progressing cSDH with mass effect [11, 18, 29, 55].

Experts have referred to cSDH in the elderly as a sentinel event of the end-of-life period due to the increased prevalence, delayed diagnosis, complex medical comorbidities and medication regimens, and significantly elevated morbidity and mortality of an often-recurrent surgical condition [2, 4, 14, 18, 36, 39, 41]. Indeed, recurrence of cSDHs after surgery occurs in up to 39% of patients. At least 20% of these patients will have poor neurological outcomes requiring long-term healthcare assistance and mortality is reported in up to 32% of patients within the first postoperative year [4, 5, 14, 36, 49, 62].

**Fig. 1:**
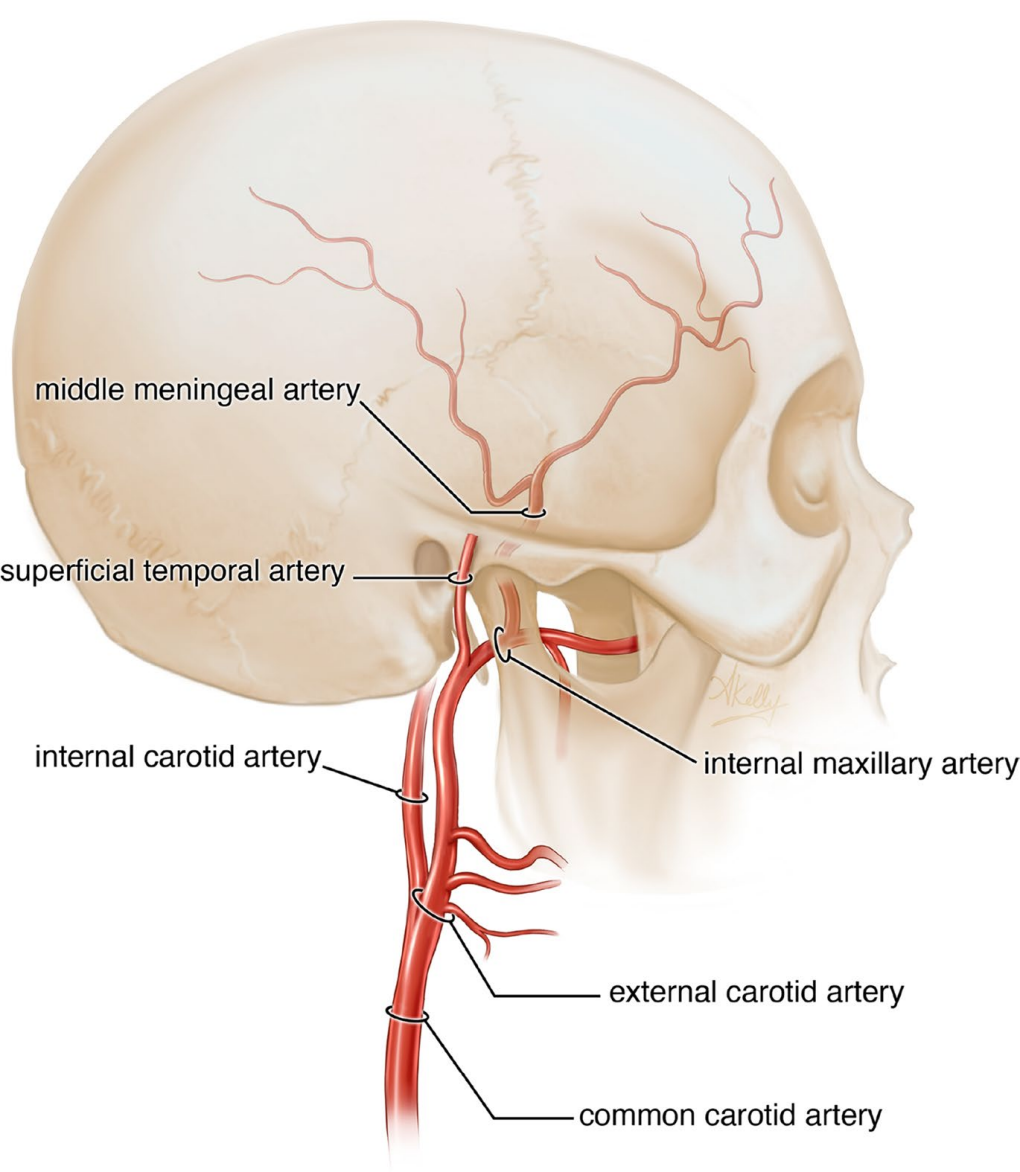
Schematic representation of pertinent vasculature off common carotid artery

In recent years, the conventional twist drill burr hole surgery for hematoma evacuation has given place to sophisticated new methods with a focus on recurrence prevention rather than simple evacuation. The middle meningeal artery is a branch distal to the external carotid artery (Fig. 1). Middle meningeal artery embolization (MMAE) is a minimally invasive, endovascular intervention growing in popularity to address these challenges by directly treating the underlying pathophysiology of cSDHs [11–13, 52]. However, methodology and implementation of MMAE for cSDH remains highly variable in current clinical practice. We provide a review on the current evidence supporting MMAE for cSDH with an emphasis on outcomes in elderly patients and the promising future directions of this well tolerated and highly effective procedure.

## Methods

### Literature search

The authors performed a comprehensive PubMed search of the literature without date restriction. The following search phrases were used: subdural hematoma, chronic subdural hematoma, middle meningeal artery embolization, and MMA embolization. A search for current clinical trials was obtained from ClinicalTrials.gov without date or study location restrictions using the following search criteria: “chronic subdural hematoma” for disease/condition and “middle meningeal artery embolization” for intervention.

### Terminology

Heterogeneity in terminology for timing and context of MMAE exists. As such, we used “primary” and “upfront” when MMAE was used as the sole intervention with standard medical care. We used “adjunct” when MMAE was used in addition to surgery and standard medical care. Additional descriptors were used for adjunct MMAE if used before surgery (“preoperative”), after surgery but before first recurrence (“prophylactic”), or after recurrence with or without additional surgery (“rescue”). These designations were based on initial group assignments in a respective study.

## Results

Our literature search initially yielded 299 publications, which were filtered to exclude those with a focus on acute SDH, SDH secondary to malignancy, and pediatric age group. Clinical studies on MMAE for cSDH were selected from highly cited articles or high-impact neurosurgical journals and included 7 case series, 16 retrospective studies, and 8 prospective studies evaluating the efficacy of primary (*n* = 20) and/or adjunct (*n* = 22) MMAE. One case series and one retrospective study were specifically designed to investigate primary and adjunct MMAE outcomes in elderly patient populations up to age 90 (Table 1). Fifteen systematic reviews with or without a meta-analysis on MMAE for cSDH published between 2019 and 2023 were additionally identified and reviewed.

**Table 1:** Selected list of notable publications on middle meningeal artery embolization for chronic subdural hematomas at time of writing. Systematic reviews and meta-analyses are excluded from this list. Primary intervention indicates MMAE was performed without surgical evacuation. Adjunct intervention includes all MMAE performed in surgical patients, including prophylactic embolization after initial surgery and rescue embolization at recurrence after initial surgical eva7cuation

Author	Year	No. patients	MMAE timing	Category	Title
Catapano et al.	2023	80	Primary	Retrospective	Middle meningeal artery embolization associated with reduced chronic subdural hematoma volume and midline shift in the acute postoperative period
Lam et al.	2023	36	Adjunct	Prospective	The efficacy of postoperative middle meningeal artery embolization on chronic subdural hematoma—a multicentered randomized controlled trial
Liebert et al.	2023	50	Adjunct	Retrospective	Embolization of the middle meningeal artery vs. second surgery-treatment response and volume course of recurrent chronic subdural hematomas
Liu et al.	2023	53	Primary Adjunct	Retrospective	Time and influencing factors to chronic subdural hematoma resolution after middle meningeal artery embolization
Orscelik et al.	2023	144	Primary	Retrospective	Middle meningeal artery embolization without surgical evacuation for chronic subdural hematoma: a single-center experience of 209 cases
Orscelik et al.	2023	51	Adjunct	Retrospective	Middle meningeal artery embolization combined with surgical evacuation for chronic subdural hematoma: a single-center experience of 75 cases
Salah et al.	2023	145	Adjunct	Retrospective	Middle meningeal artery embolization as a perioperative adjunct to surgical evacuation of nonacute subdural hematomas: an multicenter analysis of safety and efficacy
Seok et al.	2023	9	Primary	Case series	Middle meningeal artery embolization for chronic subdural hematoma in elderly patients at high risk of surgical treatment
Sioutas et al.	2023	44	Primary Adjunct	Retrospective	Middle meningeal artery embolization for subdural hematoma: an institutional cohort and propensity score-matched comparison with conventional management
Weinberg et al.	2023	99	Primary Adjunct	Retrospective	Middle meningeal artery embolization for membranous versus nonmembranous subdural hematomas: a retrospective and multicenter cohort study
Carpenter et al.	2022	250	Adjunct	Retrospective	Middle meningeal artery embolization with subdural evacuating port system for primary management of chronic subdural hematomas
Mohamed et al.	2022	15	Primary	Prospective	Middle meningeal artery embolisation for chronic subdural haematomas: the first prospective UK study
Nia et al.	2022	4274	Primary Adjunct	Retrospective	Trends and outcomes of primary, rescue, and adjunct middle meningeal artery embolization for chronic subdural hematomas
Onyinzo et al.	2022	132	Primary Adjunct	Retrospective	Efficacy and mid-term outcome of middle meningeal artery embolization with or without burr hole evacuation for chronic subdural hematoma compared with burr hole evacuation alone
Salih et al.	2022	187	Adjunct	Retrospective	Reduced recurrence of chronic subdural hematomas treated with open surgery followed by middle meningeal artery embolization compared to open surgery alone: a propensity score-matched analysis
Saway et al.	2022	100	Adjunct	Case series	Subdural evacuation port system and middle meningeal artery embolization for chronic subdural hematoma: a multicenter experience
Tanoue et al.	2022	15	Primary	Case series	The short-term outcome of middle meningeal artery embolization for chronic subdural hematoma with mild symptom: case series
Al-Mufti et al.	2021	16	Primary Adjunct	Prospective	Middle meningeal artery embolization using combined particle embolization and n-BCA with the dextrose 5% in water push technique for chronic subdural hematomas: a prospective safety and feasibility study
Catapano et al.	2021	231	Primary	Retrospective	A propensity-adjusted comparison of middle meningeal artery embolization versus conventional therapy for chronic subdural hematomas
Gomez-Paz et al.	2021	23	Primary	Case series	Upfront middle meningeal artery embolization for treatment of chronic subdural hematomas in patients with or without midline shift
Kan et al.	2021	138	Primary Adjunct	Retrospective	Middle meningeal artery embolization for chronic subdural hematoma: a multi-center experience of 154 consecutive embolizations
Khorasanizadeh et al.	2021	45	Primary	Case series	Morphological changes in chronic subdural hematomas following upfront middle meningeal artery embolization: sequence, timing, and association with outcomes
Nia et al.	2021	191	Primary	Retrospective	Middle meningeal artery embolization for chronic subdural hematoma: a national database study of 191 patients in the United States
Petrov et al.	2021	10	Primary Adjunct	Prospective	Endovascular treatment of chronic subdural hematomas through embolization: a pilot study with a non-adhesive liquid embolic agent of minimal viscosity (Squid)
Schwarz et al.	2021	41	Adjunct	Case series	Perioperative prophylactic middle meningeal artery embolization for chronic subdural hematoma: a series of 44 cases
Joyce et al.	2020	121	Primary Adjunct	Retrospective	Middle meningeal artery embolization treatment of nonacute subdural hematomas in the elderly: a multiinstitutional experience of 151 cases
Ng et al.	2020	46	Adjunct	Prospective	Middle meningeal artery embolization as an adjuvant treatment to surgery for symptomatic chronic subdural hematoma: a pilot study assessing hematoma volume resorption
Link et al.	2019	49	Primary Adjunct	Case series	Middle meningeal artery embolization for chronic subdural hematoma: a series of 60 cases
Ban et al.	2018	72	Primary Adjunct	Prospective	Middle meningeal artery embolization for chronic subdural hematoma
Kim et al.	2017	43	Adjunct	Prospective	Embolization therapy for refractory hemorrhage in patients with chronic subdural hematomas

Nineteen current clinical trials were identified with seven US trials, 11 non-US trials, and one international trial. Represented non-US countries included Canada, China, France, Germany, Iran, the Netherlands, Spain, and Sweden. Thirteen trials included the investigation of primary MMAE, and 15 trials included the investigation of adjunct MMAE (Table 2).

**Table 2:** Current registered clinical trials on middle meningeal artery embolization listed on ClinicalTrials.gov at time of writing

Sponsor/site	NCT identifier	Country	Status	Trial design
Chinese University of Hong Kong	NCT04500795	China	Not yet recruiting (starts in 2024)	Intervention Adjunct MMAE with LES for residual or recurrent cSDH (> 10 mm) on serial CT scans following initial burr hole or craniotomy evacuation for symptomatic cSDH Primary outcome Percent volume change of recurrent hematoma on serial CT scans Functional outcomes Neurological exam only Other details Age 18 + Non-randomized, single-center design Comparator: surgical evacuation
Academisch Medisch Centrum—Universiteit van Amsterdam (AMC-UvA)	NCT04511572	Netherlands	Active	Intervention Adjunct MMAE with PVA within 72 h following burr hole evacuation for symptomatic cSDH Primary outcome # patients requiring reoperation for recurrence at 8, 16, and 24 weeks after discharge Functional outcomes Neurological exam Assessments: mRS, MOCA, mNIHSS, Markwalder score, SF-36, EQ-5D-5L, ALDS, iMCQ, and iPCQ Other details Age 50–90 Randomized, single-center design Comparator: surgical evacuation
Weill Medical College of Cornell University	NCT03307395	USA	Complete/halted (2018, *n* = 6)	Intervention Primary/upfront MMAE with particle embolic agent for symptomatic cSDH not requiring immediate surgical evacuation Primary outcome Change in size of SDH, change in neurological status by exam at 1 day, 2 weeks, and 6 weeks post-MMAE Functional outcomes Neurological exam only Other details Age 18–90 Prospective intervention cohort only
University of Manitoba	NCT04923984	Canada	Active	Intervention Primary/upfront or adjunct MMAE with unspecified embolic agent for cSDH Primary outcome Recurrence on CT at 3 months Functional outcomes Neurological exam only Other details Age 18 + Prospective intervention cohort only Minimal eligibility criteria—unclear symptom requirements for inclusion
Atlantic Health System	NCT04095819	USA	Unknown	Intervention Primary MMAE with particle or liquid embolic agents for symptomatic cSDH not requiring immediate surgical evacuation Primary outcome Change in hematoma size at 6 months Functional outcomes Neurological exam only Other details Age 18 + Comparator: surgery (burr hole or craniotomy) Neurological status by exam followed after interventions
University of Manitoba	NCT04750200	Canada	Active	Intervention Primary/upfront or adjunct MMAE with PVA or liquid embolic agents for symptomatic cSDH (all intervention groups) within 48 h of surgical evacuation (adjunct intervention group) Primary outcome Recurrence within 90 days Functional outcomes Neurological exam mRS (pre-morbid 0–2 for inclusion) Other details Age 18 + Randomized Comparator: conventional treatment (surgery or medical management)
Dartmouth-Hitchcock Medical Center	NCT04270955	USA	Active	Intervention Primary/upfront or adjunct MMAE for symptomatic and asymptomatic cSDH Primary outcome Radiographic resolution at 3, 6, and 12 months Functional outcomes Neurological exam NIHSS Other details Age 18 + Randomized, single-center design Comparator: conventional treatment (observation, SEPS, burr hole, craniotomy)
Mashhad University of Medical Sciences	NCT04574843	Iran	Active	Intervention Primary/upfront MMAE with LES (Onyx, Squid, Phil) for symptomatic or medically-refractory asymptomatic cSDHs Primary outcome Volume reduction on CT or MRI at 60 days post-procedure Functional outcomes Neurological exam only Other details Age 18 + Prospective interventional cohort only Clear GCS exclusion criteria (≤ 8)
Washington University School of Medicine	NCT04065113	USA	Active	Intervention Primary/upfront or adjunct MMAE with PVA for minimally symptomatic new (primary group) or recurrent (adjunct) non-cSDH Primary outcome # patients with recurrence/refractory SDH on CT at 1 day, 7–10 days, 30 days, and 90 days # patients requiring repeat surgical evacuation Functional outcomes Neurological exam Assessments: mRS, NIHSS Other details Age 18 + Non-randomized, single-center Comparators: medical management, surgical evacuation (burr hole, craniotomy)
Balt USA	NCT04410146	USA (AZ, CA, CO, FL, GA, KS, MD, NE, NJ, PA, RI, TN, UT, WA, WV), France, Germany, Spain	Active	Intervention Primary/upfront and adjunct MMAE with Squid LES for symptomatic non-acute SDH not requiring immediate surgical evacuation Primary outcome Treatment failure within 180 days as defined by (1) residual or re-accumulation, (2) re-operation or surgical rescue, or (3) new major disabling stroke, myocardial infarction, or death from any neurological cause Functional outcomes Neurological exam Assessments: mRS (pre-morbid 0–1 for inclusion), neurocognitive battery assessment (HVLT-R, COWAT, animal naming, trail making test), EQ-5D-5L, NIHSS Other details Age 30 + Randomized, multicenter, international design
Region Skane	NCT05267184	Sweden	Active	Intervention Primary/upfront MMAE with LES for symptomatic non-acute SDH not requiring immediate surgical evacuation Primary outcome Reoperation rate Functional outcomes Neurological exam Assessments: mRS (pre-morbid 0–2 for inclusion) and EQ-5D at 3 and 12 months Other details Age 18–89 Randomized, multicenter design Comparator: surgical evacuation (burr hole, small craniotomy) with subdural or subgaleal drain Clear GCS inclusion criteria (> 13)
University Hospital, Montpellier	NCT04742920	France	Active	Intervention Primary/upfront or adjunct MMAE with Onyx LES within 72 h of randomization/surgery for symptomatic non-acute SDH Primary outcome Recurrence rate within 90 days Functional outcomes Neurological exam Assessments: mRS (pre-morbid 0–3 for inclusion), EQ-5D, Barthel Index Other details Age 18 + Randomized, multicenter design Comparators: conservative/medical management, surgery
Unfallkrankenhaus Berlin	NCT05327933	Germany	Active	Intervention Adjunct MMAE with PVA or coils + Onyx LES within 72 h of burr hole evacuation for symptomatic non-acute SDH Primary outcome Recurrence of same baseline volume or requiring reoperation within 3 months Functional outcomes Neurological exam Assessment: mRS Other details Age 18 + Randomized, single-center design
Hospital Universitari Vall d’Hebron Research Institute	NCT05220826	Spain	Active	Intervention Adjunct MMAE with Onyx, Squid, Phil, and Libro LESs within 72 h of burr hole evacuation ± subdural drain for symptomatic non-acute SDH Primary outcome Recurrence, symptomatic or radiographic, within 6 months Functional outcomes Neurological exam Assessment: mRS Other details Age 18 + Randomized, multicenter design Comparator: surgery—burr hole evacuation ± subdural drain
Cerenovus, Part of DePuy Synthes Products, Inc	NCT04816591	USA (NY, WV)	Active	Intervention Primary/upfront and adjunct MMAE with TRUFILL n-BCA for symptomatic non-acute SDH Primary outcome Recurrence or reoperation within 180 days Functional outcomes Neurological exam Assessment: mRS (pre-morbid 0–3 for inclusion) Other details Age 18–90 Randomized, multicenter design Comparators: conventional treatment (surgery, medical)
University Hospital, Brest	NCT05374681	France	Active	Intervention Primary/upfront or adjunct MMAE with cyanoacrylates for non-acute SDH Primary outcome Recurrence within 6 months by composite endpoint: (1) symptomatic, (2) reoperation, or (3) remaining or re-accumulation on CT Functional outcomes Neurological exam Assessment: mRS (pre-morbid 0–3 for inclusion), EQ-5D-5L, Barthel Index Other details Age 18 + Randomized, multicenter design Comparators: conventional treatments alone (surgery, medical)
Augusta University	NCT04272996	USA	Active	Intervention Adjunct MMAE with Onyx LES within 72 h of surgery (burr hole, small craniotomy ± subdural drain) for symptomatic non-acute SDH Primary outcome Recurrence within 3 months Functional outcomes Neurological exam Assessment: mRS (pre-morbid < 5 for inclusion) Other details Age 18–90 Randomized, single-center design Comparator: surgery alone (burr hole or small craniotomy ± subdural drain)
Medtronic Neurovascular Clinical Affairs	NCT04402632	USA (AL, AZ, CA, CO, FL, GA, IL, IN, IA, KY, MA, MI, MO, NY, NC, OH, OK, OR, PA, SC, TX, UT, WA, WI)	Active	Intervention Primary/upfront or adjunct MMAE with Onyx LES for non-acute SDH Primary outcome Recurrence or progression requiring re-intervention within 90 days Functional outcomes Neurological exam Assessment: mRS (pre-morbid 0–3 for inclusion) Other details Age 18–90 Randomized, multicenter design Comparators: conventional treatment (surgery alone, medical/observation alone)
Assistance Publique—Hôpitaux de Paris	NCT04372147	France	Unknown	Intervention Adjunct MMAE with unclear embolic agent for symptomatic, recurrent cSDH within 7 days of burr hole surgical evacuation Primary outcome Recurrence within 6 months Functional outcomes Neurological exam Assessment: mRS (pre-morbid 0–3 for inclusion) Other details Age 18 + Randomized, multicenter design Comparators: surgery alone Includes patients with higher risk for treatment failure excluded from other trials

## Discussion

### Pathophysiology, age, and role of the middle meningeal artery

**Fig. 2:**
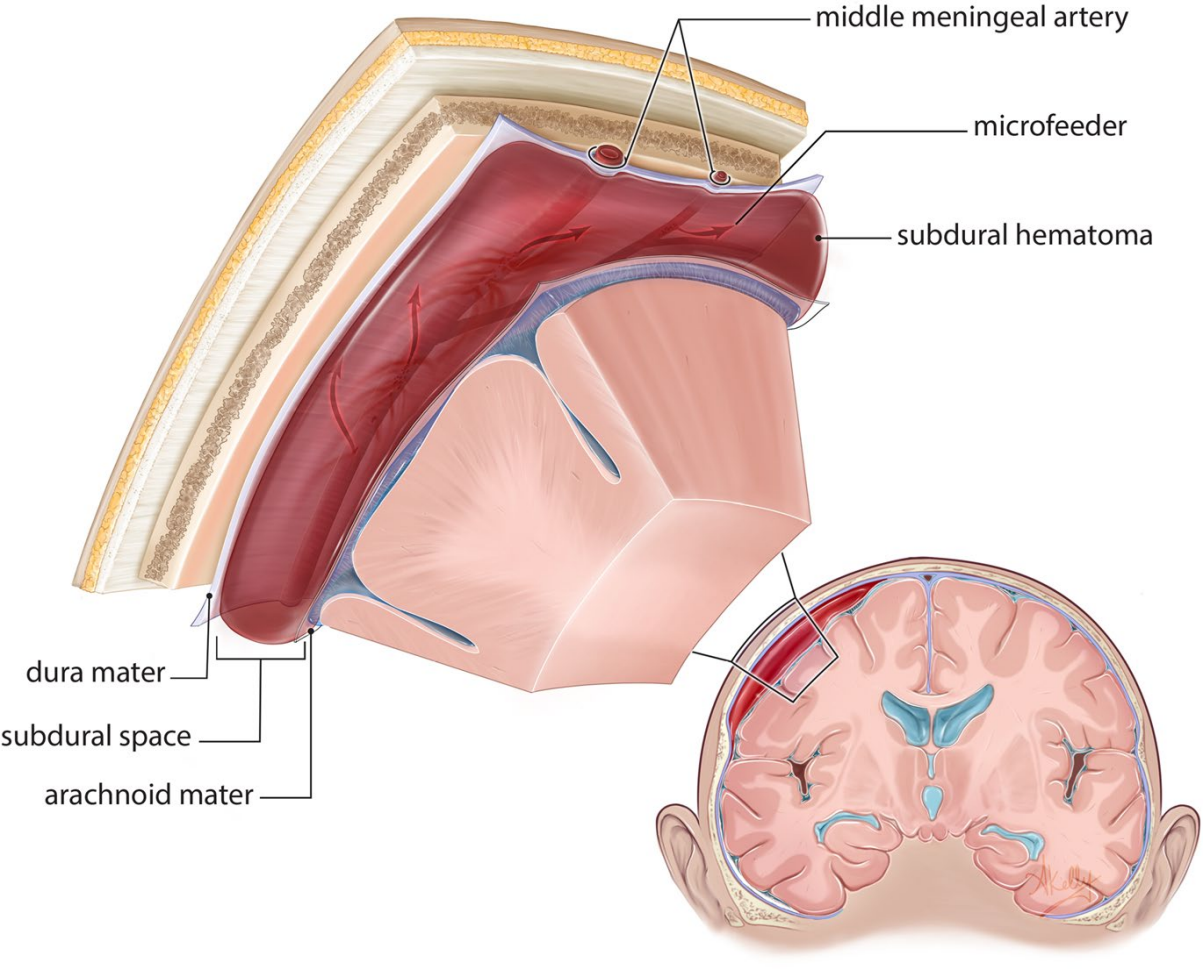
Schematic representation of subdural hematoma

Our understanding of the underlying pathophysiology of cSDH has undergone substantial evolution in the last decade. By definition, cSDH is an extra-axial collection of blood and related products encapsulated by an outer vascularized and an inner non-vascularized membrane in the subdural potential space [11, 15, 68]. This encapsulated collection is formed and expanded by a self-perpetuating cycle of inflammation, hypervascularization, exudation, and rebleeding initiated by inciting injury to the innermost dural layer [11, 15, 29, 68]. Shear stress injury disrupts the loose connectivity of dural border cells and activates a pro-inflammatory and pro-angiogenic response cascade resulting in neomembrane and microcapillary formation [15, 21, 24, 64]. These neocapillaries form preferentially in the outer membrane with anastomotic channels directly connecting to middle meningeal artery (MMA) perforating branches [11, 15, 52]. Their fragile structure with fenestrated basement membranes, absent smooth muscle layers, and numerous gap junctions makes them prone to leaking into the intramembranous collection space and producing chronic microhemorrhages [11, 15, 17]. In response, fibrinolytic activity is stimulated and additional pro-inflammatory and pro-angiogenic signals further sustain cycles of membrane formation and expansion, microvessel formation, and microhemorrhage [15, 16, 21, 24, 64]. Growth of the cSDH ensues for as long as accumulation of blood products outpaces physiologic reabsorption (Fig. 2) [11, 15].

Age over 65 years is the single greatest risk factor for development of cSDH. Brain atrophy is a known age-related phenomenon that results in altered tension mechanics along the dura and creates a potential space for extra-axial collections to develop. Additionally, anticoagulation and antiplatelet medication use is prevalent among older patients and polypharmacy in the setting of multiple medical comorbidities may further increase the risk of medication-induced coagulopathy [2, 4, 11, 14, 36, 39, 41]. Another significant factor is the increased risk of ground level falls and other age-related trauma in this patient population. Finally, Weigel et al. described multiple age-dependent cellular and molecular alterations in the immune and angiogenic pathways of cSDH pathophysiology that create a more favorable setting for its formation [68].

Overall, cSDHs are the product of a self-sustained process of chronic inflammation and angiogenesis that result in extra-axial collections within a dual-membrane capsule supplied by the MMA [11, 68]. Age is a multifactorial risk factor [4, 11, 14, 68]. Surgical evacuation involves the removal of the extra-axial collection and decompression of the underlying brain parenchyma, but surgery alone may not sufficiently disrupt the underlying pathophysiology [11, 48, 62]. Pharmacologic interventions targeting this process including dexamethasone, atorvastatin, angiotensin-converting enzyme inhibitors, selective COX-2 inhibitors, and tranexamic acid have yielded variable and non-definitive results [11, 55]. MMAE, however, is a promising, minimally invasive intervention that will disrupt further cSDH formation and expansion by occluding the MMA perforating branches supplying the leaky neocapillaries, the main source of repetitive microhemorrhages [6, 11, 15, 26, 52].

### MMA embolization for chronic subdural hematoma: outcomes and key considerations

MMAE for cSDHs is still an active area of clinical research. However, many studies are suggesting a safe and efficacious role for MMAE as a minimally invasive adjunct or alternative procedure. A selected list of important neurosurgical studies on MMAE for cSDH is included in Table 1 [3, 5, 7–9, 19, 22, 25–27, 31–34, 37, 40–46, 50, 51, 54, 56, 58, 60, 65, 69].

#### Overall outcomes

Across all published studies to date, the most common outcomes of interest include recurrence rate, re-intervention or re-operation rate, mortality rate, procedure-related complication rate, and length of hospital stay and/or intensive care unit stay [1, 12, 20, 23, 35, 53, 63, 67]. MMAE has been shown to significantly reduce recurrence rates, prevent hematoma enlargement, and reduce SDH size with minimal mortality or procedure-related complications [11]. A recent systematic review by Martinez-Perez et al. reported a pooled recurrence rate of 6.7% for primary and adjunct MMAE with a complication rate of 6% [35]. Similarly, a large meta-analysis in 2021 by Ironside et al. reported a recurrence rate of 4.8% and a re-operation rate of 4.4%, which were significantly reduced compared to their medically managed comparison group (21.5% and 16.4%, respectively) [20]. Mortality is reported at 1% or less across these systematic studies. Numerous additional systematic reviews reinforce these general findings for MMAE with recurrence and treatment failure in less than 10% of patients and favorable neurological outcomes in greater than 75% of patients [1, 12, 20, 23, 35, 53, 63, 67].

#### Adjunct MMAE

**Fig. 3:**
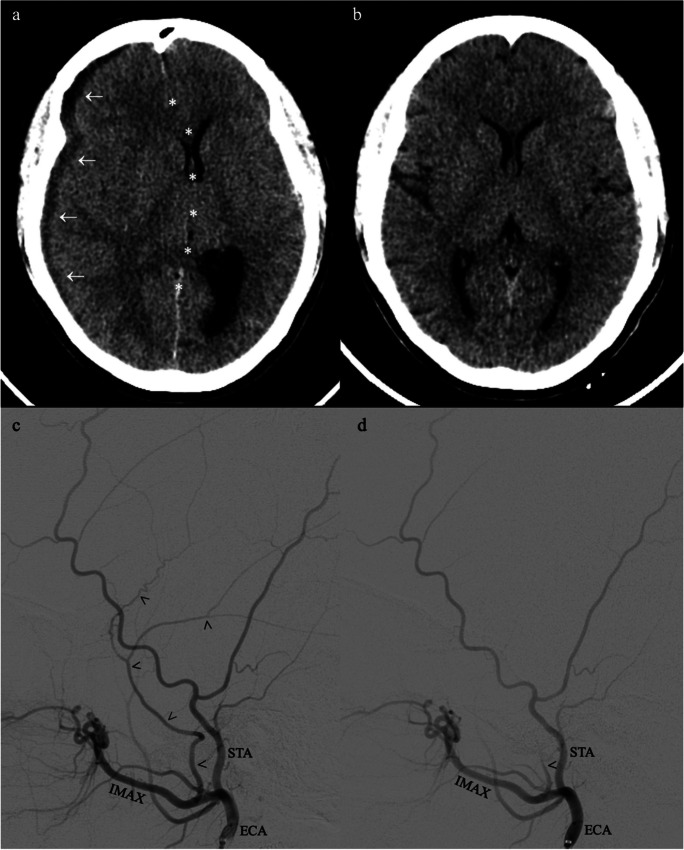
Newer treatment of chronic subdural hematoma. Before (**a**/**c**) and after (**b**/**d**) treatment involving surgical evacuation and middle meningeal embolization. Axial non-contrasted computer tomography of sample patient (**a**/**b**). Digital subtraction angiography, lateral view, right external carotid injection of sample patient (**c**/**d**). Chronic subdural hematoma (arrows), right-to-left midline shift (stars), middle meningeal artery (arrow heads)

Ample evidence exists to support MMAE for cSDH as an adjunct intervention to prevent recurrence after primary surgical evacuation (Fig. 3, Table 1). Link et al. provided the first large case series in 2019 of 60 cases across 49 patients to include adjunct MMAE as prophylactic or rescue intervention with a recurrence and reoperation rate of 8.9% and 3 non-procedure-related mortalities [33]. In 2021, Schwarz et al. reported a similar recurrence rate (9.1%) with prophylactic adjunct MMAE after subdural evacuating port system (SEPS) or craniotomy, and reoperation was only needed for SEPS-evacuated cSDH recurrence (4.5%) [56]. While these results suggested superior avoidance of repeat surgery with initial craniotomy and adjunct MMAE, Saway et al. supported the less invasive approach of SEPS and adjunct MMAE in their retrospective series of 100 patients with a 6.6% recurrence rate, 2% reoperation rate, and overall favorable functional outcomes (mRS < 2) in the majority of patients at final follow-up [54].

The most recently published retrospective studies on adjunct MMAE for cSDH have further strengthened the findings of these early, smaller studies and broadened the clinical context in which MMAE has been successfully employed. Joyce et al. retrospectively analyzed adjunct MMAE following SEPS, burr hole evacuation, or craniotomy specifically among elderly patients across 15 academic centers with recurrence rate of 5.4% [22]. Further studies reported similar findings in their retrospective analysis with recurrence rates as low as 5.4% even among significantly older patient groups with added medical complexity and anticoagulation use [7, 25, 32, 34, 43, 44, 50, 51, 60, 69]. Overall, MMAE is an adjunct intervention that can significantly reduce recurrence and need for surgical rescue among operative symptomatic cSDH patients regardless of the selected method for surgical evacuation with low mortality, morbidity, and complication rates.

#### Primary MMAE

Substantial attention has also been devoted to MMAE as a primary, solo intervention for cSDH. It was typically considered for patient populations at higher surgical risk or in those with asymptomatic or minimally symptomatic subdural collections. Many of the aforementioned studies also included primary MMAE groups in their analysis without significant differences in recurrence rates, reoperation rates, functional outcomes, or mortality and complications [22, 25, 33, 34, 43, 44, 60, 69]. In the most recent, single-center retrospective series of 144 patients by Orscelik et al. on primary MMAE, 72.8% of patients had greater than 50% reduction in hematoma size with 13.8% requiring reintervention, 8.4% mortality rate, and 49.3% with improved functional outcomes [45]. In comparison, the adjunct MMAE series from the same researchers reported a greater proportion (93.3%) of patients achieving greater than 50% hematoma reduction with less need for reintervention (6.7%), lower mortality (2%), and more patients with improved functional outcomes (78.4%) [44]. Differences in patient cohorts included antiplatelet/anticoagulant use, symptom severity, and complexity of medical comorbidities.

These results, along with the prior literature and Sattari et al.’s systematic review and meta-analysis on primary MMAE demonstrated that less favorable surgical candidates with less severe symptoms may still gain benefits from primary MMAE in reducing recurrence and reintervention rates while improving functional outcomes. However, adjuvant MMAE may provide greater reduction in recurrence and reintervention outcomes if the patient is an appropriate surgical candidate [8, 9, 19, 22, 25, 26, 33, 34, 41–43, 45, 53, 58, 60, 65, 69].

#### Particle versus liquid embolic agents

Multiple embolic agents are available for MMAE and are broadly categorized as particle or liquid embolic agents. Particle agents include polyvinyl alcohol (PVA). Liquid embolic agents include ethylene vinyl alcohol copolymer (Onyx®, Medtronic Neurovascular, Irvine, CA, USA; Squid®, Balt, Montmorency, France) and *N*-butyl-2-cyanoacrylate (NBCA; TruFill®, Cerenovus, Irvine, CA, USA) [66]. Questions remain over whether one type of embolic agent provides superior results over another. A key challenge is heterogeneity in embolic agents used in individual studies. Ku et al.’s recent systematic review suggested that Onyx® may provide better recurrence and reintervention reduction while particle agents combined with coil embolization may provide better clinical outcomes [30]. Two systematic reviews reported recurrence and reoperation rates as low as 3% without significant difference between liquid and particle embolic agents but a trend toward superior outcomes for primary MMAE with liquid embolic agents [38, 61]. Abdollahifard et al., however, demonstrated similar outcomes with particle embolic agents in their 2022 systematic review and meta-analysis [1]. To date, no definitive differences in radiographic and clinical outcomes based on liquid and particle embolic agent choice have been demonstrated in the context of MMAE for cSDH [57].

#### Neurological and functional outcomes

Recent studies have emphasized neurological and functional outcomes by using quantitative, evidence-based measurements such as the modified Rankin Scale (mRS), the NIH Stroke Scale (NIHSS), the Barthel Index, and systems for assessing quality of life [8, 22, 31, 44, 45, 50, 51, 54, 60]. mRS appeared frequently in the MMAE literature and emphasized motor function outcomes and functional independence on a numerical scale from 0 to 6 with a favorable outcome typically considered a score of 2 or less [47]. MMAE studies have assessed mRS changes as either proportion of patients in the favorable category or median change in scoring with categorization as improved, unchanged, or worsened [8, 22, 31, 44, 45, 50, 51, 60].

Adjunct MMAE can significantly improve functional outcomes as assessed by mRS with most studies reporting improvement in at least 70 to 75% of patients to the favorable category [8, 22, 31, 44, 45, 50, 51, 60]. Although previously discussed individual studies on primary MMAE suggested significant improvement in mRS functional outcomes in selected patients, a recent systematic review by Sattari et al. suggested that primary MMAE may not significantly reduce the proportion of patients with less favorable outcomes (mRS > 2) over conventional therapy (i.e., surgical evacuation) [53]. Although mRS is commonly used, alternative systems for assessing functional outcomes in this patient population may provide better prognostic information but have not been adequately compared to date. Thus, further, specific investigation on functional outcome improvements after MMAE in its various contexts is needed.

### Perspectives on MMA embolization for elderly patients

cSDH is a neurosurgical condition of utmost significance among patients aged 65 years and older for whom MMA embolization is an appealing, minimally invasive, endovascular option to improve long-term outcomes [4, 11]. As previously discussed, important factors contributing to cSDH development are prevalent in this population, including age-related diffuse cerebral atrophy, use of antiplatelet and anticoagulant medications, and increased frequency of minor trauma [2, 14, 18, 36, 39, 41]. Additionally, elderly patients represent a special population in operative decision-making as they often have more medical complexity from comorbid disease(s), have a higher risk of complications from general anesthesia, are more susceptible to postoperative infections, and have increased risk of deconditioning with prolonged postoperative immobility [10, 28, 36, 59].

Overall, the available evidence for primary and adjunct MMAE was predominantly derived from elderly patients with typical median and/or average ages of 65–78 years [3, 5, 7–9, 19, 22, 25–27, 31–34, 37, 40–46, 50, 51, 54, 56, 58, 60, 65, 69]. Joyce et al. specifically investigated outcomes for primary and adjunct MMAE among elderly (65–79 years) and advanced elderly (80 years and older) patients in a multi-institutional series of 121 patients. Majority of patients in both age groups were male and had multiple cardiovascular comorbidities, and antiplatelet and anticoagulant use at presentation was reported in 49% of elderly and 69% of advanced elderly patients. Their results demonstrated radiographic stability or improvement of non-acute SDHs—including cSDHs—in the majority of elderly and advanced elderly patients (91% and 98%, respectively) without elevated reintervention rates (4.6% and 7.8%, respectively) or mortality rates (8.6% and 3.9%, respectively) [22].

MMAE as a primary or adjunct intervention for elderly patients with cSDHs is presented as safe and efficacious to date [22, 58, 65]. Primary and adjunct intervention reduce recurrence and reintervention rates significantly in comparison to the recurrence and reintervention rate of up to 39% with conventional management. Additionally, the addition of MMAE appears to reduce the proportion of patients with poor neurological and functional outcomes. These improvements are seemingly accomplished without significant change in complication rates and may also be reducing the 1-year mortality rate from 32% with conventional management to less than 10% [12, 20, 23, 53, 63, 67].

### Prospective results and ongoing clinical trials

A key limitation of even the strongest studies is limited validation with prospective results directly comparing MMAE to conventional management. Kim reported the first prospective results of adjunct MMAE in 20 patients which demonstrated no significant outcome differences to conventional management and described predictive factors of hematoma recurrence [27]. In 2018, Ban et al. reported prospective, non-randomized trial outcomes for 27 asymptomatic patients treated with primary MMAE and 45 symptomatic patients treated with adjunct MMAE. No hematoma re-accumulation was reported in the primary MMAE group, one case of re-accumulation (2.2%) was reported in the adjunct MMAE group, and recurrence rate of 27.5% was reported in a historical conventional treatment group. Their prospective results in larger cohorts support that primary and adjunct MMAE significantly reduce recurrence and reintervention rates (*p* = 0.001) without significant increase in complications (0% vs. 4.3%, *p* = 0.182) [5]. The pilot and feasibility studies of Ng et al. and Al-Mufti et al. were consistent with the prior literature results for adjunct MMAE as well [3, 40]. Two small, non-US pilot studies (10 and 15 patients, respectively) and one non-US prospective study (36 patients) similarly support prior findings for primary and adjunct MMAE with liquid embolic agents [31, 37, 46]. The latter study also reported significant improvement in functional outcome with all adjunct MMAE treated patients achieving mRS 0 or 1 at 3 months while only 53% of the surgery control group achieved the same outcome [31]. Preliminary results from a prospective randomized trial being conducted at our institution comparing surgery alone versus surgery + MMAE are suggesting improved outcomes in the combination treatment group (Table 2).

Multiple clinical trials are currently being conducted to provide level one evidence on the efficacy of primary or adjunct MMAE and are summarized in Table 2. All current clinical trials are investigating the same post-procedure outcomes frequently reported by prior case-level and retrospective studies. Additionally, fourteen trials incorporate some form of assessment of functional and quality of life outcomes beyond serial neurological exams, most commonly mRS.

Thirteen trials involve investigation of primary MMAE, and fifteen trials involve investigation of adjunct MMAE. Of the primary MMAE trials, three trials will perform primary MMAE for asymptomatic or minimally cSDHs (Dartmouth-Hitchcock, USA, NCT04270955; Mashhad University, Iran, NCT04574843; Washington University, USA, NCT04065113). Most adjunct MMAE trials involve performing MMAE during the same hospitalization and within 48–72 h of surgical evacuation of the symptomatic cSDH. One trial in France (NCT04372147) will perform adjunct MMAE up to 7 days postoperative. Multicenter trials sponsored by Medtronic (EMBOLISE trial, NCT04402632), Balt (NCT04410146), and Cerenovus (MEMBRANE trial, NCT04816591) are also ongoing to support the efficacy of their respective liquid embolic agents: Onyx®, Squid®, and TruFill® n-BCA.

Two parallel trials are currently investigating prophylactic adjunct MMAE with the Onyx® LES: the Augusta University trial (NCT04272996) and the Medtronic-sponsored EMBOLISE trial (NCT04402632). Results from the single-center Augusta University trial demonstrate the potential promise of the eventual results of the multicenter EMBOLISE trial with its similar design and outcomes of interest. Both will provide needed prospective evidence for the efficacy of adjunct MMAE for cSDH with Onyx® LES, including for elderly and octogenarian patients.

### Future directions and key research questions

High-quality, prospective evidence supporting MMAE for cSDHs will soon be available to inform and unify current clinical practice. Nevertheless, additional questions in optimizing the approach for effectively applying MMAE in the overall management of cSDHs remain to guide further study. Key questions include the timing of MMAE, particularly as an adjunct to surgical evacuation; the optimal strategy for asymptomatic or minimally symptomatic cSDHs; embolic agent selection; prophylactic bilateral embolization for unilateral cSDHs; and the role of concomitant medical adjuvant therapies, such as steroids, statins, and anti-inflammatory therapies.

## Conclusion

In summary, cSDH is mainly a disease of the older population. Its traditional treatment option involved surgical evacuation. Unfortunately, the outcomes following standalone surgery have remained undesirable, resulting in significant morbidity and mortality. Newer treatment options including embolization of the MMA are advancing the practice of neurosurgery to enhance patient care by decreasing hematoma recurrence and improving neurological outcomes.
